# Interleukin-6 as an early marker for fat embolism

**DOI:** 10.1186/1749-799X-4-18

**Published:** 2009-06-13

**Authors:** R Yoga, JC Theis, M Walton, W Sutherland

**Affiliations:** 1 Department of Orthopaedic Surgery, University of Otago, Dunedin, New Zealand; 2 Department of Medicine, University of Otago, Dunedin, New Zealand

## Abstract

**Background:**

Fat Embolism is a complication of long bone fractures, intramedullary fixation and joint arthroplasty. It may progress to fat embolism syndrome, which is rare but involves significant morbidity and can occasionally be fatal. Fat Embolism can be detected at the time of embolization by transoesophageal echocardiography or atrial blood sampling. Later, a combination of clinical signs and symptoms will point towards fat embolism but there is no specific test to confirm the diagnosis. We investigated serum Interleukin-6 (IL-6) as a possible early marker for fat embolism.

**Methods:**

An animal study was conducted to simulate a hip replacement in 31 adult male Sprague Dawley rats. The procedure was performed under general anesthesia and the animals divided into 3 groups: control, uncemented and cemented. Following surgery and recovery from anaesthesia, the rats allowed to freely mobilize in their cages. Blood was taken before surgery and at 6 hours, 12 hours and 24 hours to measure serum IL-6 levels. The rats were euthanized at 24 hours and lungs removed and stained for fat. The amount of fat seen was then correlated with serum IL-6 levels.

**Results:**

No rats in the control group had fat emboli. Numerous fat emboli were seen in both the uncemented and cemented implant groups. The interleukin levels were raised in all groups reaching a peak at 12 hours after surgery reaching 100 pg/ml in the control group and around 250 pg/ml in the uncemented and cemented implant groups. The IL-6 levels in the control group were significantly lower than any of the implant groups at 12 and 24 hours. At these time points, the serum IL-6 correlated with the amount of fat seen on lung histology.

**Conclusion:**

Serum IL-6 is a possible early marker of fat embolism.

## Introduction

Fat embolism occurs when fat and marrow content from bone gains access to the circulation and becomes trapped in the capillaries of the lungs or other organs [1]. Bone marrow in the long bones of mature adults contains up to 92% of fat [2]. The fat is released following skeletal trauma [3,4] or from manipulation of the medullary canal during hip and knee arthroplasty [5]. Fat emboli in the lungs trigger an inflammatory process leading to hemorrhage and leakage of proteins into the alveoli [6].

Inflammatory mediators have also been implicated in the lung changes following fat embolism [7,8] such as IL-6, CD-11b expression, elastase and s-E-selectin [9]. One study showed that IL-6 and TNF-alpha were elevated in cases of multiple fractures [8]. Syrbu concluded that following fat embolism broncho-alveolar lavage contained inflammatory mediators [10].

IL-6 is one of the most important cytokine in the acute inflammatory phase [11]. It is also one of the mediator that is released very early in an injury process [12]. At the moment, there is no specific blood test that can be used to detect fat embolism. Diagnosis is mainly by clinical features and supported by a few laboratory tests. This often results in delayed treatment which can be detrimental to the patient. Having a reliable marker for fat embolism would be of great clinical benefit and the aim of our study was to determine whether IL-6 was such a marker.

An animal model using Sprague Dawley rats was used for this study. Many studies have utilized rats as a model for fat embolism [13-17]. Most of them used external infusion of fatty acids to simulate fat embolism. However bone marrow cells are a significant part of the material embolized and in order to reproduce the clinical situation more closely we chose to embolize the animal's own medullary content rather than injecting fatty acids. The medullary cavity of Sprague Dawley rats has been shown to contain around 5% of fat cells [18]. Our animal model for fat embolism is described in the methodology section. It has been shown that rats have a heart circulation that closely resembles that of humans making them suitable to study the effects of embolism on the cardio repiratory system [19]. The rat animal model was chosen as it allowed us to carry out a surgical procedure generating fat embolislm, measure serum levels of IL-6 and provided lung biopsy samples to confirm that embolism had actually occurred and also to quantify the amount of fat deposited in the lungs. For those reasons the Sprague Dawley rat was used as an animal model in this study.

The objective of this study was to assess whether the serum concentration of an inflammatory marker could be correlated to the extent of inflammation secondary from fat embolism. The ultimate aim would be to use the marker as an early diagnostic tool for a condition (FES) notorious for late detection. Although there are many inflammatory markers, we selected IL-6 as a marker for a few reasons. Firstly the ELISA kit for measuring IL-6 required 0.5 mls of blood for each measurement. As inflammatory cytokines are elevated in hemorrhagic shock [20,21] we wanted to reduce the amount of blood taken by limiting our study to IL-6. Removal of up to 7.5% (1.5 mls in our 500 gm rats) of the total circulating blood volume is considered to be safe [22] and in order to avoid stressing the animals and falsely raising the inflammatory cytokines we selected to study only one inflammatory marker. Secondly, IL-6 is an inflammatory marker that is activated in the early phase of the acute inflammatory response and therefore would allow early detection and treatment of fat embolism. Finally, we had to limit ourselves to one marker as the rat model did not allow us to draw enough blood to study multiple markers.

### Procedure

Thirty one adult male Sprague Dawley rats were used. They were divided into 3 groups: control (n = 9), uncemented implant (n = 10) and cemented implant (n = 12). Prior approval from the institutional animal ethics committee was obtained. These rats were housed in standard solid floor cages with wood shaving litter. The animals were induced using inhalational halothane anesthesia with a mixture of oxygen. Depth of anesthesia was assessed by pedal reflex. After each animal was anaesthetized, it was placed in a supine position and the skin over both knee joints was shaved and disinfected. A skin incision was made over the flexed knee joint. A medial parapatella incision allowed retraction of the patella laterally, exposing the distal femur.

There were 3 different groups:

#### i) Controls

A 2 mm drill perforated the articular cartilage at the superior end of the inter-condylar notch, taking great care not to breach the medullary canal. The medial parapatella wound was closed using Vicryl sutures and the skin with staples. This was repeated on the contralateral side.

#### ii) uncemented implant group

**Figure 1:**
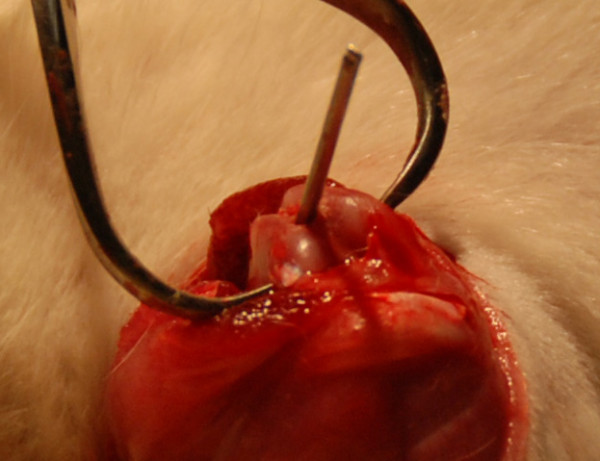
**Kirschner wire being advanced into the femoral canal**.

This group had the same surgical approach as the controls. However after breaching the articular cartilage, the 2 mm drill was advanced well into the medullary canal. A 2 mm Kirchner wire (2.5 cm long) was then advanced (Figure 1) until it was flush with the joint surface. The wound was closed in the same manner as the controls. This was repeated on the contralateral side.

#### iii) cemented implant group

**Figure 2:**
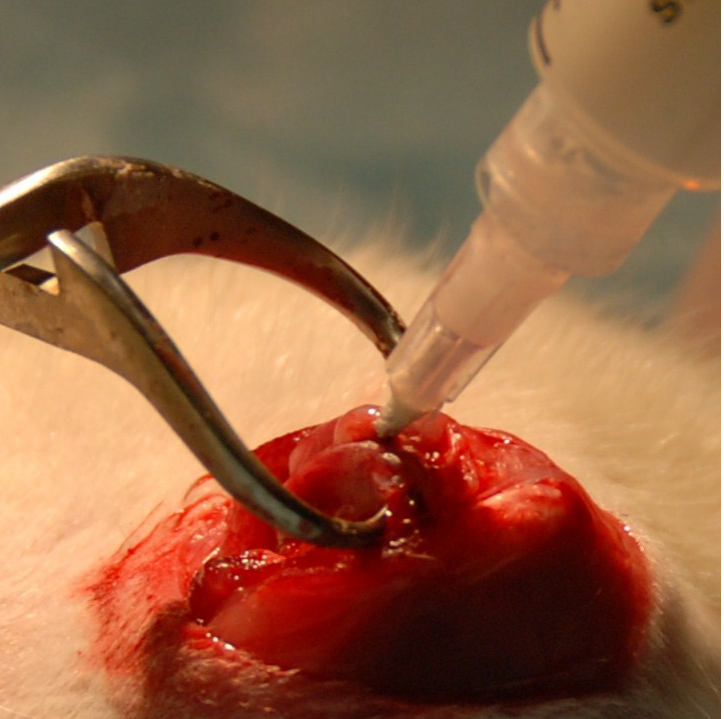
**Cement being injected into the medullary canal using a syringe**.

They had the identical approach as the uncemented implant group up to the drilling of the medullary canal. Then, a freshly mixed bone cement (CMW 1, DePuy International Ltd, England) was pressurized into the canal using a syringe and modified needle (Figure 2). A 2 mm Kirchner wire (2.5 cm long) then advanced into the canal until it was flush with the joint surface. The extruded cement was removed. The wound was closed in the same manner as the controls. This was repeated on the contralateral side.

The same surgeon performed all surgery. The three groups were alternated sequentially until all the rats were operated. Blood (0.5 mls) was taken from a tail vein before surgery, at 6 hours and at 12 hours while the animals were briefly anaesthetised. At 24 hours, all the rats were euthanized. Intracardiac blood was then taken immediately after death. A midline chest incision was made to expose the lungs and heart. The trachea was transected and the lungs expanded using formalin saline injected via the cut trachea. The right upper lobe and the left lobe were processed for histological examination. Osmium Tetroxide was used to stain fat within the tissues [23,24]. The lungs were wax-impregnated and sectioned. Finally they were counterstained with Haematoxilin and Eosin.

Slides were viewed using light microscopy with a help of a pathologist. In order not to under or over estimate the amount of embolised material, all fat globules seen in the slide were counted at 10× magnification. Subsequently, the surface area for the lung section was estimated using a 10 mm by 10 mm square grid (the grid was made up of 1 mm^2 ^boxes). The crossectional areas of the right upper lobe and the left lobs sections were determined. The density of fat emboli per 100 mm^2 ^was then calculated.

The blood collected was initially centrifuged at 4000 revolutions per minute for 4 minutes. The serum was then isolated and stored in a minus 80 degrees fridge until all the samples had been collected. On the day of ELISA analysis, the samples were thawed to room temperature. The serum was then processed following the recommendations of the IL-6 kit manufacturer (R&D Systems).

All the reagents (which were stored at minus 20 degrees Celsius) were brought to room temperature. The 96 well ELISA plate was filled with 50 μl of assay diluents in each well. Each sample was processed in duplicate with the final result calculated as the mean of both values. The first 2 rows (8 wells in each row) were filled up with 50 μl of either the standard or control samples. There were 7 standards of known IL-6 values, which ranged from the lowest to the highest measurable IL-6 concentration that was provided by the manufacturer (62.5 pg/l to 4000 pg/l) and the control was a single sample of known concentration (given by the manufacturer) that was used to ensure quality control. The subsequent 10 rows were filled with 50 μl of test serum to each well and these too were processed in duplicate. All plates were incubated for 2 hours. They were then aspirated and washed 5 times using a plate washer. Subsequently 100 μl of conjugate was added to each well and incubated for 2 hours. It was then aspirated and washed with a buffer solution 5 times. Finally 100 μl of substrate solution was added and protected from light for 30 minutes. Then 100 μl of stop solution was applied to each well. The optical density was then read at 450 nm on an Omron spectrophotometer. The reading was printed out and the duplicates averaged out. A standard curve was drawn using the values obtained from the 7 standards. The control sample ensured that the quality readings obtained was within the set limits. Finally, the values for the test samples were worked out from the standard curve.

We postulated that more extensive fat embolism occurred in the uncemented and cemented implant groups. We used the Analysis of Variance (ANOVA) to see if there were any statistical differences between the three groups with regards to the amount of fat. Subsequently, we applied Fisher's Least Significant Difference (LSD) as a post hoc test to study if there were any differences between these three groups. We used p < 0.05 as the confidence interval. We also postulated that the IL-6 levels would be higher if there was more fat embolism seen, therefore a comparison was calculated with the IL-6 levels at the various time points. ANOVA was used with LSD as a post hoc procedure to see if there was any difference in the 3 groups studied in the four different time points.

Finally, we wanted to see if we could correlate the fat embolism to the measured IL-6 levels. For this, we initially combined all the three different groups and compared the IL-6 levels at the three different time points (6 hours, 12 hours and 24 hours) with the amount of fat embolism seen. Correlation of the number of fat emboli and IL-6 levels at 6 hours, 12 hours and 24 hours irrespective of the three groups was carried out using the Spearmans Rank Correlation test. This test gives a Spearman Correlation Coefficient between -1 and +1. A value closer to +1 will indicate a direct positive correlation. All the tests completed were using the Statistical Package for Social Sciences (SPSS) Version 11 for Windows.

## Results

Out of the 31 rats in this study 3 rats died during surgery. All of them belonged to the cemented implant group and they were excluded from the study.

**Figure 3:**
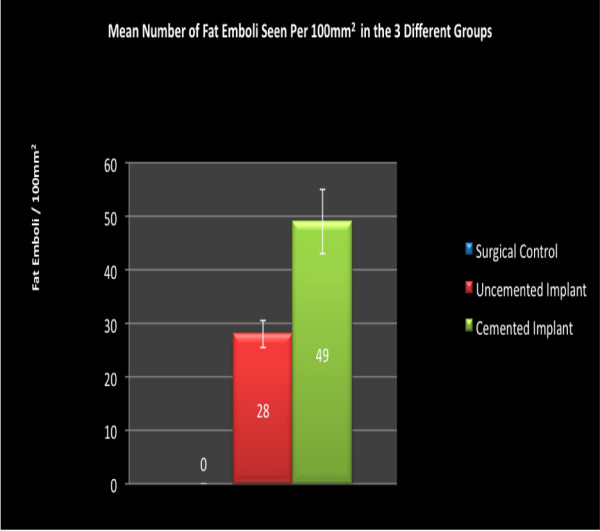
**Fat emboli seen in the 3 different groups**.

**Figure 4:**
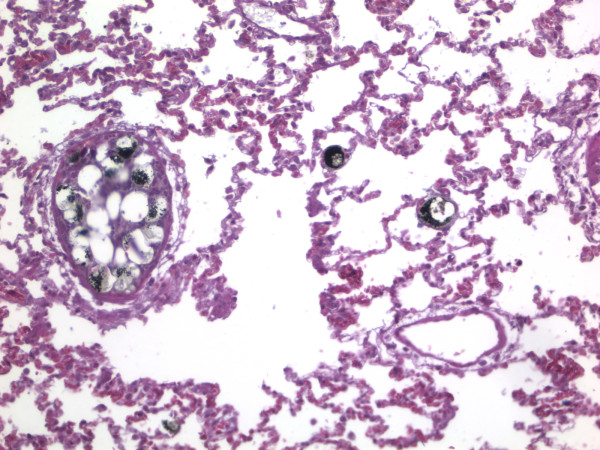
**Presence of fat (stained black) in the lungs of the rat**. There are numerous inflammatory cells and red blood cells seen outside the capillaries.

Figure 3 shows the fat emboli density in the 3 different groups. There were no emboli seen in the control group. Both the uncemented (p < 0.034) and cemented implant groups (p < 0.001) had a significantly higher number of fat emboli compared to the control group. There were also a significantly higher number of emboli (p < 0.040) in the cemented compared to the uncemented implant group. In the slides where there was fat embolism, there was a clear aggregation of inflammatory cells and hemorrhage in the alveoli (Figure 4).

**Figure 5:**
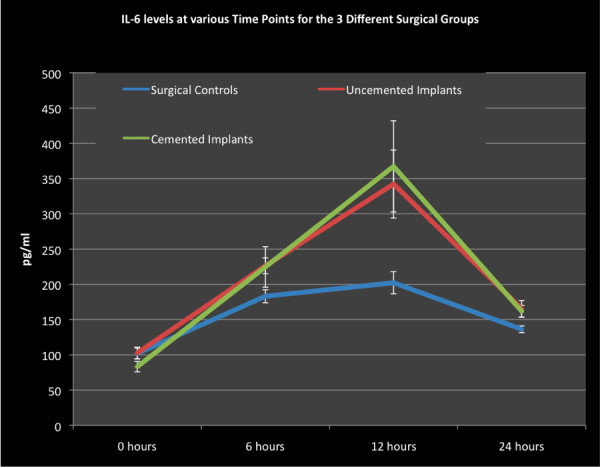
**Serum Interleukin-6 levels plotted at 4 different time points**.

The IL-6 levels were raised in the three groups reaching a peak at 12 hours after surgery (Figure 5). At this time point, levels had risen by a 100 pg/ml for the control and around 250 pg/ml for the implant groups. Subsequently, all three groups demonstrated a fall in IL-6 levels up to 24 hours. There were significant changes between the groups at 12 hours (p < 0.049) and at 24 hours (P < 0.025). The IL-6 levels in the control group were significantly lower than in the uncemented implant group at 12 hours (p < 0.046) and 24 hours (p < 0.025). IL-6 levels in the control group were also significantly lower at 12 hours (p < 0.024) and 24 hours (p < 0.013) compared to the cemented implant group. There was no significant difference in the IL-6 levels between the uncemented and cemented implant group at all time points. Although the IL-6 levels dropped after 12 hours in all 3 groups, it did not reach the pre operative levels at 24 hours.

**Figure 6:**
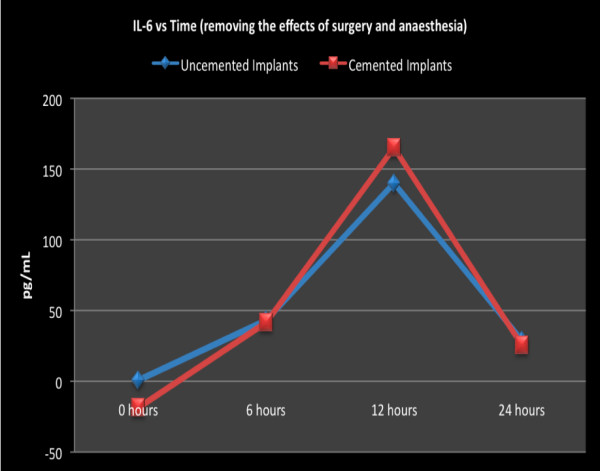
**Serum IL-6 values against time between the cemented and uncemented group after values from the control group were subtracted from the corresponding values**.

From the results above, it is clear that the surgical approach to the femur or anesthesia or both had a part to play in the rise of IL-6. In an attempt to remove this influence, we subtracted the IL-6 levels measured in the control group from the levels measured in the implant groups for all respective time points. This gave an estimation of Il-6 rise without the influence of surgical approach or anesthesia. Figure 6 shows that there is still a peak at 12 hours.

The most likely cause of the raised IL-6 levels in the implant groups is fat embolism as the only difference between them and the control group was the fat seen on lung histology. The correlation coefficient at 6 hours between IL-6 and the number of emboli seen was 0.292. At 12 and 24 hours the correlation coefficient was 0.494 and 0.405 respectively. This indicates that there was a positive correlation between fat embolism and IL-6. This correlation was stronger at 12 and 24 hours.

## Discussion

The receptor for IL-6 is found on many cell surfaces including resting normal T-cells, activated normal B-cells, myeloid cell lines and others. IL-6 stimulates acute phase reaction that is responsible for fever, increased erythrocyte sedimentation rate and activation of clotting cascades, all of which are seen in established fat embolism syndrome.

IL-6 is sensitive to tissue damage [25] and local levels of IL-6 may be higher than systemic levels [26-29]. In our study only systemic levels of IL-6 were measured and they could be an overspill of the local IL-6 [29] and we therefore assumed that systemic IL-6 levels correlate with local IL-6 levels. Inflammation in the lungs probably is thought to be due to the toxic effects of the free fatty acids released from the breakdown of fat. Histology of the lungs in this study confirmed the presence of inflammation at the time of death. (Figure 3).

One study claimed that the inflammatory changes in the lungs were immune mediated [7] whereas another concluded that fat emboli are not the only cause of FES [30]. It is interesting that although fat embolism will occur in almost all cases of long bone fractures, only a few cases will develop fat embolism syndrome. One possible explanation is the production of inflammatory mediators. Evidence has shown that IL-6 is also responsible for adipose tissue metabolism, lipoprotein lipase activity and hepatic triglyceride secretion [31]. There is a polymorphism of the IL-6 gene that will result in abnormalities in IL-6 transcription rate. Experimental studies in humans comparing the activity of this gene have shown that patients with polymorphism of the IL-6 gene are prone to lipid abnormalities [32]. Although this was not demonstrated by these authors, this abnormal IL-6 transcription could well lead to an abnormal reaction to fat emboli in the lungs and the development of a full blown fat embolism syndrome.

Various types of orthopaedic operations lead to an increase in IL-6 serum levels [28,33]. This explains why in the present study there was a rise in IL-6 levels in all 3 groups as the surgery involved some degree of muscle injury to get access to the knee joint. However, although the surgical approach was the same in the 3 groups, the variation in IL-6 was significantly different and we have shown that fat embolism demonstrated histologically must be a major factor.

There was significant variability in IL-6 levels post surgery within each group of animals. This variability has also been shown by Minetto in his study if IL-6 levels after hip surgery [34]. It is possible that different individuals release different quantities of IL-6 when presented with the same stimuli. Apart from that, he noted that the upward slope of the IL-6 curve was related to the duration of surgery. He also found that higher IL-6 levels were associated with higher postoperative fever but without evidence of differences in postoperative problems, time to mobilize, or duration of stay in hospital. Postoperative infection was also associated with higher IL-6 levels [35]. In our study, all animals were operated on in a small animal operating theatre using clean surgical techniques. The surgical time varied between 10 to 15 minutes and the same surgical protocol was used in all cases by one single operator.

Inflammatory cells are responsible for the release of IL-6. If it was released solely as a result of tissue trauma, it should have increased straight after surgery and peaked at 6 hours. However in our study the peak was at 12 hours that makes tissue trauma from the surgical procedure unlikely. We have shown that release of IL-6 was due to fat embolism and secondary lung tissue damage. This effect was noted up to 24 hours. It is likely that emboli in the lung trigger an inflammatory reaction, which takes time to become established before IL-6 levels increase. This may explain why there was no correlation between IL-6 rise and the amount of fat emboli at 6 hours.

The significant correlation shown at 12 hours and 24 hours demonstrates that IL-6 rise is related to fat embolism. In this study, the correlation was 0.494 and 0.405 at 12 and 24 hours respectively

This is an animal model and although we tried to reproduce a surgical procedure as closely as possible, the findings cannot be automatically transferred to clinical practice. IL-6 is only one of the inflammatory markers and there are surely others involved in the pathogenesis of fat embolism.

We have shown, using an animal fat embolism model, that there are lung changes which correlate with IL-6 serum levels which makes us believe that IL-6 may be one of the early markers of fat embolism. Further research is required to validate the use of IL-6 as a reliable marker to detect fat embolism in a clinical setting.

## Competing interests

The authors declare that they have no competing interests.

## Authors' contributions

YR carried out the surgery, interpretation of data and preparing the manuscript.

JCT gave the idea for this research planning and finally in drafting the manuscript.

MW was involved with the surgery, interpretation of data and critically apprising the manuscript.

WS carried out the ELISA test.
